# Chronic Mexiletine Administration Increases Sodium Current in Non-Diseased Human Induced Pluripotent Stem Cell-Derived Cardiomyocytes

**DOI:** 10.3390/biomedicines12061212

**Published:** 2024-05-29

**Authors:** Giovanna Nasilli, Arie O. Verkerk, Molly O’Reilly, Loukia Yiangou, Richard P. Davis, Simona Casini, Carol Ann Remme

**Affiliations:** 1 Department of Experimental Cardiology, Amsterdam University Medical Center, University of Amsterdam, Heart Center, Meibergdreef 9, 1105 AZ Amsterdam, The Netherlandsa.o.verkerk@amsterdamumc.nl (A.O.V.);; 2 Amsterdam Cardiovascular Sciences, Heart Failure & Arrhythmias, 1105 AZ Amsterdam, The Netherlands; 3 Department of Medical Biology, Amsterdam University Medical Center, University of Amsterdam, Meibergdreef 9, 1105 AZ Amsterdam, The Netherlands; 4 Department of Anatomy and Embryology, Leiden University Medical Center, Albinusdreef 2, 2300 RC Leiden, The Netherlands; 5 The Novo Nordisk Foundation Center for Stem Cell Medicine (reNEW), Leiden University Medical Center, Albinusdreef 2, 2300 RC Leiden, The Netherlands

**Keywords:** ion channels, cardiac electrophysiology, sodium current, therapy, hiPSC-CMs

## Abstract

A sodium current (I_Na_) reduction occurs in the setting of many acquired and inherited conditions and is associated with cardiac conduction slowing and increased arrhythmia risks. The sodium channel blocker mexiletine has been shown to restore the trafficking of mutant sodium channels to the membrane. However, these studies were mostly performed in heterologous expression systems using high mexiletine concentrations. Moreover, the chronic effects on I_Na_ in a non-diseased cardiomyocyte environment remain unknown. In this paper, we investigated the chronic and acute effects of a therapeutic dose of mexiletine on I_Na_ and the action potential (AP) characteristics in human induced pluripotent stem cell-derived cardiomyocytes (hiPSC-CMs) of a healthy individual. Control hiPSC-CMs were incubated for 48 h with 10 µM mexiletine or vehicle. Following the wash-out of mexiletine, patch clamp analysis and immunocytochemistry experiments were performed. The incubation of hiPSC-CMs for 48 h with mexiletine (followed by wash-out) induced a significant increase in peak I_Na_ of ~75%, without any significant change in the voltage dependence of (in)activation. This was accompanied by a significant increase in AP upstroke velocity, without changes in other AP parameters. The immunocytochemistry experiments showed a significant increase in membrane Na_v_1.5 fluorescence following a 48 h incubation with mexiletine. The acute re-exposure of hiPSC-CMs to 10 µM mexiletine resulted in a small but significant increase in AP duration, without changes in AP upstroke velocity, peak I_Na_ density, or the I_Na_ voltage dependence of (in)activation. Importantly, the increase in the peak I_Na_ density and resulting AP upstroke velocity induced by chronic mexiletine incubation was not counteracted by the acute re-administration of the drug. In conclusion, the chronic administration of a clinically relevant concentration of mexiletine increases I_Na_ density in non-diseased hiPSC-CMs, likely by enhancing the membrane trafficking of sodium channels. Our findings identify mexiletine as a potential therapeutic strategy to enhance and/or restore I_Na_ and cardiac conduction.

## 1. Introduction

The cardiac sodium channel, encoded by the gene *SCN5A*, enables sodium influx into cardiomyocytes, leading to the rapid upstroke of the action potential and, as such, is essential for excitability and proper electrical conduction in the heart [1,2]. Sodium channel dysfunction occurs in various acquired and inherited cardiac conditions, including myocardial ischemia, heart failure, cardiomyopathy, long QT syndrome (type 3, LQTS3), and Brugada syndrome [3,4]. Depending on the underlying mechanism, these conditions may be associated with a decreased peak sodium current (peak I_Na_) and/or an increased late sodium current (late I_Na_) that persists throughout the action potential (AP) plateau and repolarization phase [2,5]. A decreased peak I_Na_ leads to a reduced AP upstroke velocity and cardiac conduction slowing, setting the stage for (re-entrant) arrhythmias and sudden cardiac death [4]. However, despite decades of research, there are as yet no pharmacological options to enhance peak I_Na_.

Mexiletine is a sodium channel blocker and a class Ib anti-arrhythmic drug [6]. Clinically, it is used in patients suffering from recurrent ventricular arrhythmias secondary to myocardial infarction and ischemic heart disease, particularly in cases where conventional therapies, such as beta blockers or amiodarone, are suboptimal or not well tolerated [7,8,9,10]. In addition, mexiletine may also block late I_Na_ and, as such, shorten AP duration in the setting of long QT syndrome [11,12]. Indeed, mexiletine has been shown to prevent ventricular tachycardia in patients with LQTS3 [11], but its effects vary between different *SCN5A* mutations [13]. Previous studies using radioligand assays have demonstrated that the in vivo administration of mexiletine for 72 h in rats increased the number of sodium channels in cardiomyocytes [14,15]. In addition, the chronic administration of mexiletine for 24–48 h has been shown to rescue the reduced peak I_Na_ associated with certain *SCN5A* mutations, most likely by acting as a pharmacological chaperone and increasing the membrane trafficking of mutant channels [16,17,18,19]. However, most of these mutation studies were performed in non-cardiac cells and employed concentrations of mexiletine that are much higher (>100 µM) than the clinical therapeutic range (plasma concentration of 0.8–2 mg/L or approximately 3–12 µM) [20], which would be expected to have significant (extra) cardiac side effects. We have previously shown that the chronic administration of a therapeutic concentration of 10 µM mexiletine was able to partly restore the reduced peak I_Na_ associated with the *SCN5A*-1795insD mutation in HEK293 cells [21]. Moreover, chronic mexiletine administration increased peak I_Na_ and AP upstroke velocity in human induced pluripotent stem cell-derived cardiomyocytes (hiPSC-CMs) from a patient carrying this mutation [21]. Interestingly, we also observed that a 48 h incubation with 10 µM mexiletine increased peak I_Na_ in HEK293 cells expressing wild-type *SCN5A* [21]. While these observations indicate that a clinically relevant concentration of mexiletine may be beneficial even in the absence of an *SCN5A* mutation, its chronic effects on I_Na_ in a non-diseased cardiomyocyte environment remain as yet unknown. In this paper, we therefore investigated the chronic and acute effects of mexiletine on I_Na_ and AP characteristics in control hiPSC-CMs.

## 2. Materials and Methods

### 2.1. Generation of hiPSC-CMs

Control hiPSCs were differentiated into hiPSC-CMs, cryopreserved and thawed as previously described [20]. In summary, glass coverslips were coated with 1:100 matrigel in DMEM-F12 (cat. no. 31331028, Thermo Fisher Scientific, Waltham, MA, USA) for 2 h at 37 °C before the thawed hiPSC-CMs were seeded at a density of 9.5 × 10^3^/cm^2^. The day after thawing and then every 2–3 days following this, the cells were refreshed with RPMI medium (cat. no. 21875034,Thermo Fisher Scientific,Waltham, MA, USA) supplemented with B27. Three days prior to the patch clamp experiments, the medium was supplemented with 5% fetal calf serum. The patch clamp and immunocytochemistry experiments were performed 8–10 days after the initial cell seeding.

### 2.2. Drug Incubation

hiPSC-CMs were incubated in a medium containing either the vehicle (H_2_0) or 10 µM mexiletine (Tocris Bioscience, Abingdon, UK) for 48 h at 37 °C. After 48 h, hiPSC-CMs were washed three times with either the RPMI/B27 medium supplemented with 5% serum (but containing no mexiletine) prior to the patch clamp experiments or with PBS prior to cell fixation (see scheme in the relevant figures). To assess the acute effect of the drug, hiPSC-CMs previously incubated for 48 h with 10 µM mexiletine or vehicle were superfused for 5 min with 10 µM mexiletine (see scheme in the relevant figures).

### 2.3. Patch Clamp Analysis

Data acquisition. Peak I_Na_ and APs were measured with the ruptured and perforated patch clamp technique, respectively, using an Axopatch 200B amplifier (Molecular Devices, San Jose, CA, USA). Voltage control, data acquisition, and analysis for I_Na_ recordings were performed with pClamp10.6/Clampfit (Molecular Devices, San Jose, CA, USA) while for AP recordings we used a custom-made acquisition software (‘Scope’, version 04.04.27) and data analysis software (‘MacDaq’, version 8.0). Borosilicate glass patch pipettes (World Precision Instruments, Sarasota, FL, USA) with a tip resistance of 2–2.5 MΩ were used. Cell membrane capacitance was determined dividing the decay time constant of the capacitive transient in response to 5 mV hyperpolarizing steps from −40 mV, by the series resistance. Series resistance and cell membrane capacitance were compensated for ≥80%. I_Na_ and APs were filtered at 5 kHz and digitized at 40 kHz. I_Na_ was measured at room temperature, while APs were recorded at 37 °C.

Sodium current. Peak I_Na_ measurements were performed using the following external solution (in mM): 130 NaCl, 20 CsCl, 1.8 CaCl_2_, 1.2 MgCl_2_, 11 D-glucose, 0.005 nifedipine, 5.0 HEPES; pH 7.4 (CsOH); and pipette solution containing (in mM): 3.0 NaCl, 133 CsCl, 2.0 MgCl_2_, 2.0 Na_2_ATP, 2.0 TEACl, 10.0 EGTA, 5.0 HEPES; pH 7.3 (CsOH). Peak I_Na_ was elicited from a holding potential of −120 mV with a cycle length of 5 s (see insets in relevant figures). Current-voltage (I-V) relationships and the voltage dependency of activation were characterized with 500 ms depolarizing pulses between −100 and +60 mV. The voltage dependency of inactivation was analyzed using a double-pulse protocol, with 500 ms prepulses ranging between −130 and −25 mV and a step to −20 mV. Peak I_Na_ was defined as the difference between the peak and steady state current at the end of each step. The current densities were calculated by dividing the current amplitude by the cell membrane capacitance. The voltage dependence of activation and inactivation curves were fitted with the Boltzmann function (y = [1 + exp{(V-V_1/2_)/*k*}]^−1^), where V_1/2_ is the half-maximal voltage of (in)activation and *k* the slope factor.

Action potentials. APs were measured using a modified Tyrode’s solution containing (in mM): 140 NaCl, 5.4 KCl, 1.8 CaCl_2_, 1.0 MgCl_2_, 5.5 D-glucose, 5 HEPES; pH 7.4 (NaOH). The pipettes were filled with (in mM): 125 K-gluconate, 20 KCl, 5 NaCl, 0.44 amphotericin-B, 10 HEPES, pH 7.2 (KOH). The APs were elicited at 1 Hz by 3-ms, ≈1.2× threshold current pulses through the patch pipette. To overcome the lack of the inward rectifying potassium current (I_K1_) in hiPSC-CMs, which limits the functional availability of I_Na_ [22], we injected an in silico I_K1_ with an I-V relationship of Kir2.1 channels [23] and a peak outward density of 2 pA/pF through a dynamic clamp, as previously described and validated in detail [24]. The resting membrane potential (RMP), AP amplitude (APA), maximal AP upstroke velocity (V_max_), and AP duration (APD) at 20, 50, and 90% repolarization (APD_20_, APD_50_, and APD_90_, respectively) were analyzed. Data from 10 consecutive APs were averaged, and the potentials were corrected for the calculated liquid junction potential.

### 2.4. Immunocytochemistry

Following 48 h treatment with either 10 µM mexiletine or vehicle (H_2_O), hiPSC-CMs were washed three times with PBS and fixed with 4% PFA for 10 min. The cells were permeabilized with 0.2% Triton-X in PBS for 10 min, followed by a 30 min blocking step in PBS containing 2% glycine, 2% bovine serum albumin (BSA), 0.2% gelatin, and 10% normal goat serum. The anti-α-actinin mouse primary antibody (cat.no. A7811, Sigma-Aldrich, St. Louis, MA, USA, dilution 1:200) and anti-Na_V_1.5 rabbit primary antibody (cat. no. ASC-005, Alomone Labs, Jerusalem, Israel, dilution 1:100) were incubated for 1 h at room temperature. After washing with PBS, the secondary anti-rabbit antibody conjugated with Alexa Fluor 488 (cat. no. A-11008, Invitrogen, Waltham, MA, USA, dilution 1:300) and anti-mouse antibody conjugated with Alexa Fluor 568 (cat. no. A-11004, Invitrogen, Waltham, MA, USA, dilution 1:300) were incubated for 1 h at room temperature. Negative controls were created under the same conditions but without primary antibodies. For quantification, four areas (regions of interest) along the cell membrane were selected in each cell, and Na_V_1.5 fluorescence intensity was analyzed in these areas using ImageJ. Two rounds of immunostaining were included in the analysis. Images were acquired using a Leica Stellaris 5 confocal microscope with a HC PL APO CS2 63×/1.40 oil objective, with 405 (DAPI), 488 (Na_v_1.5), and 568 (α-actinin) nm laser lines.

### 2.5. Statistical Analysis

The data were analyzed using Sigma Stat 3.5 (Systat Software Inc.) and GraphPad Prism version 8.4.3(686) for Windows (GraphPad Software). The values are shown as mean ± SEM. Normality was tested by the Kolmogorov-Smirnov test. An unpaired Student’s t-test or a paired Student’s t-test was used in the case of normally distributed data. A Mann-Whitney test or a Wilcoxon rank test was applied when normality and/or equal variance test failed. The statistical significance for differences in the current-voltage relations (I-V) curves was determined by performing a two-way repeated measures (RM) ANOVA, followed by Holm-Sidak test for a post hoc analysis. A one-way RM ANOVA followed by a Holm-Sidak test for post hoc analysis or a one-way RM ANOVA on Ranks (Friedman test) followed by Tukey’s test when the data were not normally distributed was used to compare multiple groups. The level of statistical significance was set to *p* < 0.05.

## 3. Results

### 3.1. Chronic Mexiletine Treatment Increases Peak I_Na_ in hiPSC-CMs

**Figure 1:**
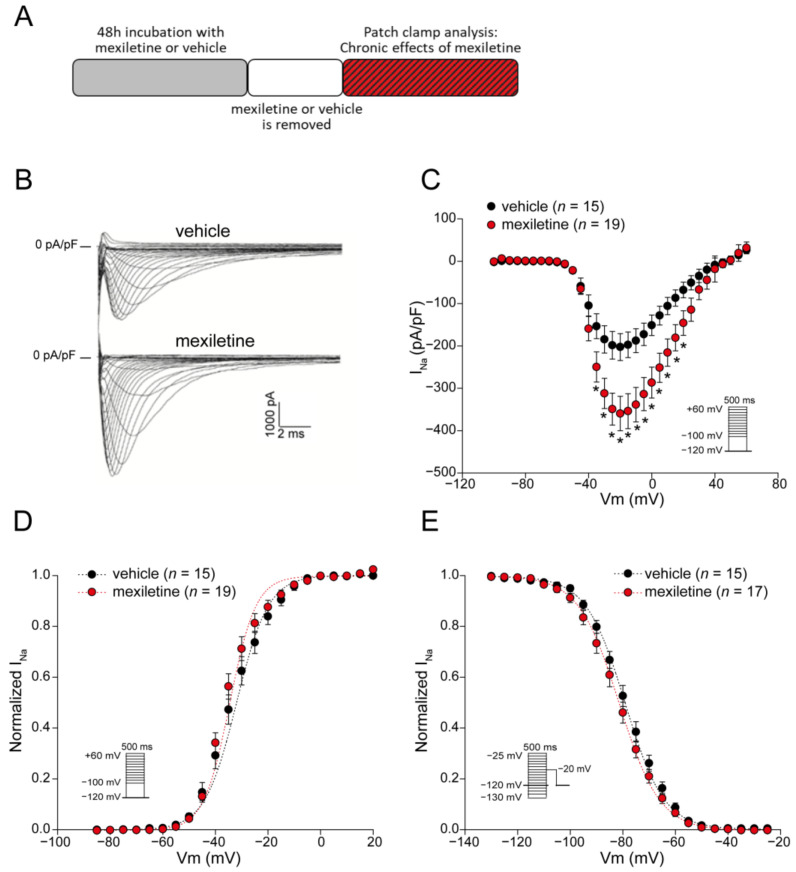
Incubation of hiPSC-CMs for 48 h with 10 µM mexiletine increases I_Na_ density. (**A**) Schematic representation of the experimental approach. (**B**) Representative I_Na_ traces for hiPSC-CMs recorded after a 48 h incubation with the vehicle or mexiletine (10 µM). Average current-voltage relationships in hiPSC-CMs (**C**) and voltage dependence of activation (**D**) and inactivation (**E**). Insets: voltage clamp protocols; *n*, number of cells; * *p* < 0.05, two-way RM ANOVA followed by a Holm-Sidak test for post hoc analysis.

**Figure 2:**
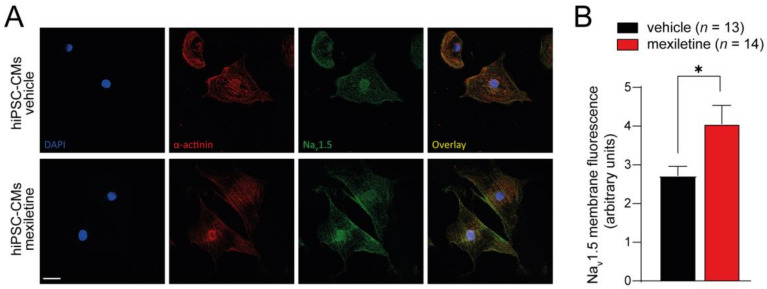
Immunostaining of hiPSC-CMs treated with vehicle or 10 µM mexiletine for 48 h. (**A**) Example images of hiPSC-CMs stained for DAPI (blue), α-actinin (red), and Na_V_1.5 (green). (**B**) Quantification of Na_V_1.5 membrane fluorescence intensity. Scale bar is 25 µm; *n* represents the number of cells analyzed. * *p* < 0.05, unpaired Student’s *t*-test.

We first assessed the chronic effect of mexiletine on peak I_Na_ in hiPSC-derived cardiomyocytes (hiPSC-CMs). The cells were incubated with 10 µM mexiletine or the vehicle for 48 h followed by the removal of the drug or vehicle (see scheme in Figure 1A). Chronic incubation with mexiletine resulted in a significant increase in the average I_Na_ density as compared to vehicle incubation (~75% increase at −20 mV: vehicle −202.7 ± 31.7 pA/pF, *n* = 15 versus mexiletine −359.6 ± 40.6 pA/pF, *n* = 19; *p* < 0.05) (Figure 1B,C). In Figure 1D,E, the effects of 48 h mexiletine incubation on the I_Na_ voltage dependence of activation and inactivation are depicted. No significant differences in the half-maximal voltage of (in)activation (V_1/2_) or the slope factor k were observed in hiPSC-CMs incubated with either mexiletine or vehicle (Table 1). Overall, these observations indicate that chronic incubation with mexiletine at a clinically relevant dose of 10 µM is capable of increasing peak I_Na_ in non-diseased hiPSC-CMs without affecting its gating properties, suggesting that it increased the membrane trafficking of Na_V_1.5. Indeed, the immunocytochemistry experiments showed a significant increase in Na_V_1.5 fluorescence along the cell membrane in hiPSC-CMs incubated with mexiletine compared to those treated with the vehicle (Figure 2).

### 3.2. Acute Administration of Mexiletine Does Not Affect Peak I_Na_ in hiPSC-CMs

**Figure 3:**
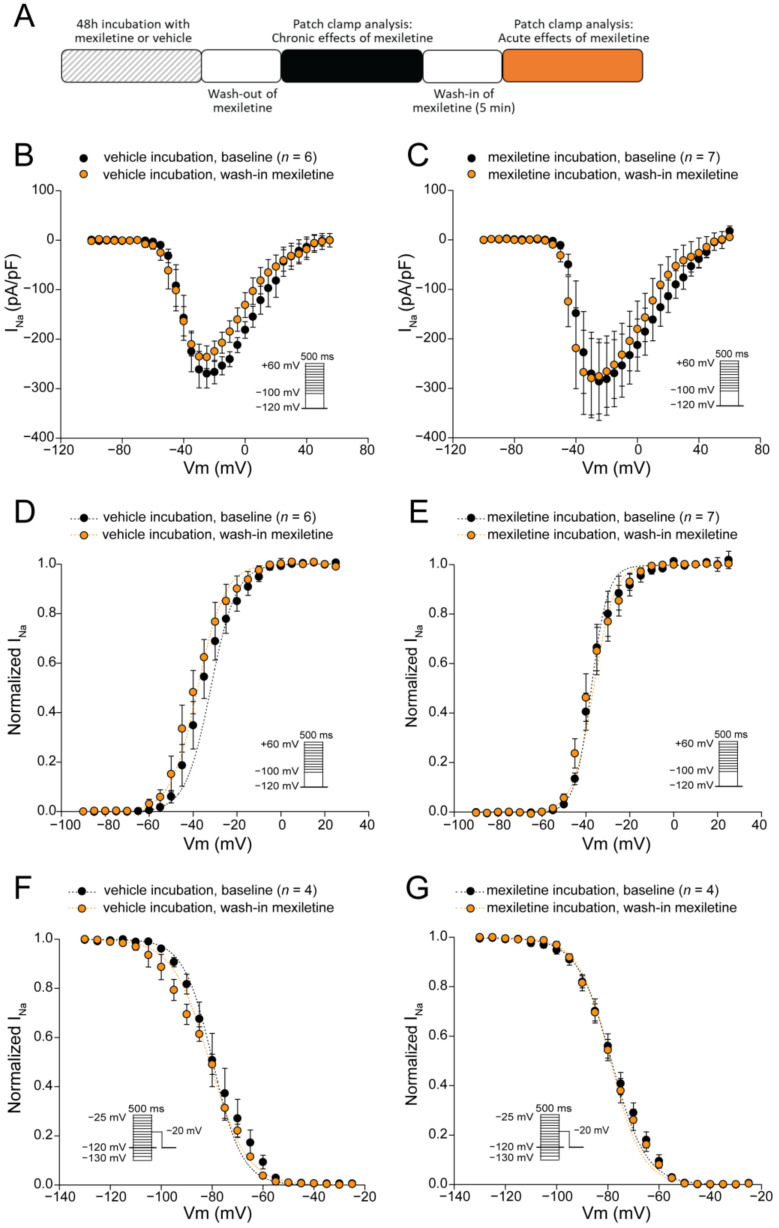
Acute effect of mexiletine on I_Na_ density and I_Na_ voltage dependence of (in)activation in hiPSC-CMs. (**A**) Schematic representation of the experimental approach. (**B**,**C**) Average sodium current-voltage relationships of hiPSC-CMs (previously incubated for 48 h with the vehicle (**B**), or mexiletine (**C**)) at baseline and after the acute (re-)administration (5 min) of 10 µM mexiletine. (**D**–**G**) Voltage dependence of activation (**D**,**E**) and inactivation (**F**,**G**) measured in hiPSC-CMs (previously incubated for 48 h with the vehicle (**D**,**F**), or mexiletine (**E**,**G**)) at baseline and after the acute (re-)administration (5 min) of 10 µM mexiletine. Insets: voltage clamp protocols; *n*, number of cells. V_1/2_ and slope factor *k* values are listed in Table 2.

In the experiments described above (presented in Figure 1), mexiletine was washed out prior to patch clamp analysis. While this approach allowed us to investigate specifically the chronic impact of the drug without its direct, acute effect on the channel, it does not reflect chronic mexiletine therapy in patients. To mimic the clinical situation where the drug is continuously present, we employed the same approach as above (i.e., 48 h of incubation with 10 µM mexiletine or the vehicle followed by wash-out), followed by the acute re-administration of 10 µM mexiletine to the bath solution (see scheme in Figure 3A) and patch clamp analysis. As shown in Figure 3B,C, the acute administration of mexiletine to hiPSC-CMs previously incubated for 48 h with either mexiletine or the vehicle did not affect peak I_Na_. Moreover, no effects were observed of the acute mexiletine administration on the voltage dependency of activation or inactivation (Figure 3D–G, Table 2). Hence, the increase in peak I_Na_ induced by chronic mexiletine incubation in hiPSC-CMs was not mitigated by the acute (re-)application of the drug.

### 3.3. Chronic Mexiletine Increases Action Potential Upstroke Velocity in hiPSC-CMs

**Figure 4:**
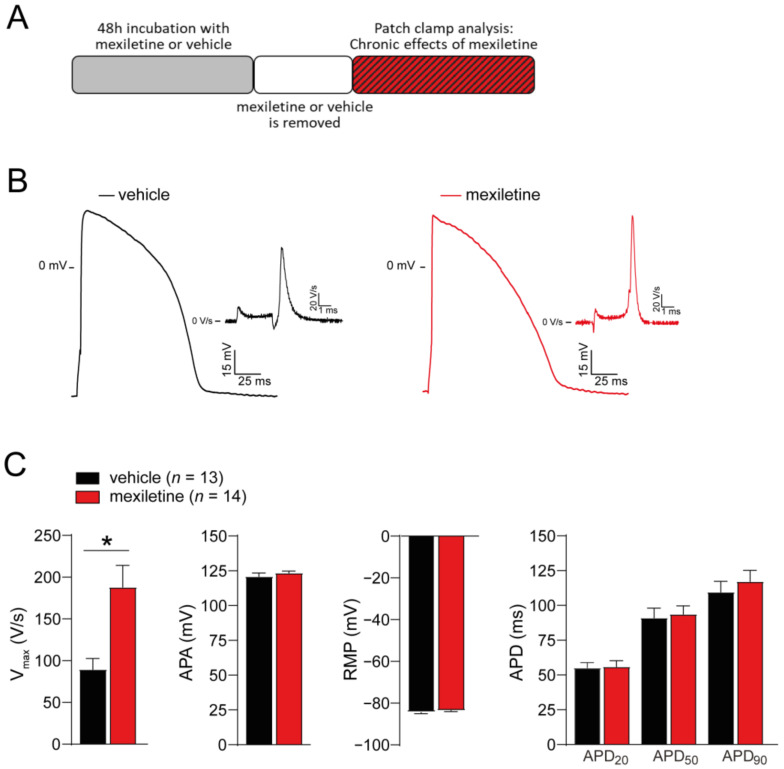
Incubation of hiPSC-CMs for 48 h with 10 µM mexiletine increases action potential upstroke velocity without affecting repolarization. (**A**) Schematic representation of the experimental approach. (**B**) Representative action potential (AP) traces of hiPSC-CMs incubated for 48 h with the vehicle or mexiletine (10 µM). Insets: first derivative of the AP upstroke. (**C**) Average values for the AP upstroke velocity (V_max_), AP amplitude (APA), resting membrane potential (RMP), and AP duration at 20%, 50% and 90% repolarization (APD_20_, APD_50_, and APD_90_, respectively), measured at a pacing frequency of 1 Hz. *n*, number of cells. * *p* < 0.05, unpaired Student’s *t*-test.

We next assessed the functional impact of mexiletine on AP properties in hiPSC-CMs, employing dynamic clamp for in silico I_K1_ injection in cells paced at 1 Hz at physiological temperature (37 °C). In line with the observed increase in peak I_Na_, the incubation of hiPSC-CMs with mexiletine for 48 h (followed by the removal of the drug; Figure 4A) significantly increased the AP upstroke velocity (V_max_; ~110% increase compared to the vehicle-incubated cells, Figure 4B,C). In contrast, no differences were observed in the AP amplitude, resting membrane potential (RMP), or AP duration (APD) at 20, 50, and 90% repolarization (APD_20_, APD_50_, and APD_90_, respectively) between hiPSC-CMs incubated with mexiletine and the cells incubated with the vehicle only (Figure 4C). Hence, the chronic treatment of hiPSC-CMs with mexiletine increased V_max_ without affecting repolarization.

### 3.4. No Acute Effect of Mexiletine on AP Upstroke Velocity in hiPSC-CMs

**Figure 5:**
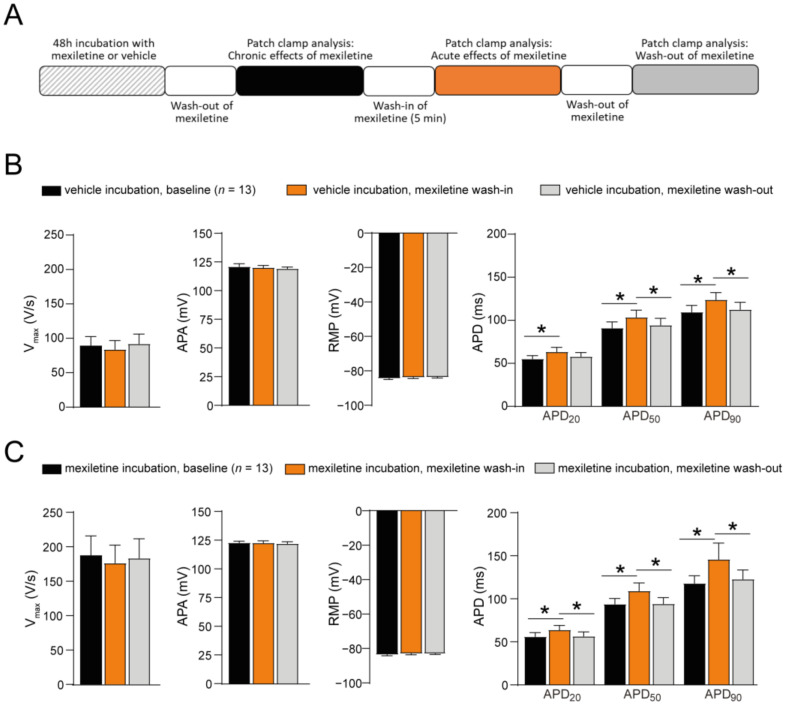
Acute effect of mexiletine on action potential properties in hiPSC-CMs. (**A**) Schematic representation of the experimental approach. (**B**) Average data for the maximal upstroke velocity (V_max_), AP amplitude (APA), resting membrane potential (RMP), AP duration at 20%, 50%, and 90% repolarization (APD_20_, APD_50_, and APD_90_, respectively) in hiPSC-CMs after a 48 h incubation with the vehicle (baseline), after 5 min of the acute administration of 10 µM mexiletine (wash-in), and after the wash-out of the drug. (**C**) Average data for V_max_, APA, RMP, APD_20_, APD_50_, and APD_90_ in hiPSC-CMs after a 48 h incubation with 10 µM mexiletine (baseline), after 5 min of the acute re-administration of 10 µM mexiletine (wash-in), and after wash-out. *n*, number of cells; * *p* < 0.05, one-way RM ANOVA followed by a Holm-Sidak test for post hoc analysis or a one-way RM ANOVA on Ranks (Friedman test) followed by Tukey’s test for post hoc analysis when the data were not normally distributed.

The chronic effects of mexiletine on AP characteristics depicted in Figure 4 were obtained in the absence of mexiletine in the bath solution. To explore the acute effects of mexiletine on AP characteristics, we assessed the effects of the acute (re-)administration of the drug to hiPSC-CMs, employing an approach similar to the one used to assess the acute effects on I_Na_ (Figure 5A). The effects of wash-out following the acute administration of mexiletine was also investigated. The acute (re-)administration of mexiletine to hiPSC-CMs previously incubated for 48 h with either mexiletine or the vehicle had no significant effect on APA or RMP (Figure 5B,C). In contrast, a small but significant increase in APD_20_, APD_50_, and APD_90_ was observed following the acute administration of mexiletine, both in hiPSC-CMs previously incubated with mexiletine as well as in those chronically incubated with the vehicle (Figure 5B,C). This prolongation of AP duration was reversible upon the wash-out of mexiletine. Importantly, the acute (re-)administration of mexiletine to hiPSC-CMs chronically incubated with either mexiletine or the vehicle had no significant effect on V_max_ (Figure 5B,C). Hence, in line with the observation that mexiletine did not acutely affect peak I_Na_, the increase in the AP upstroke velocity induced by chronic mexiletine incubation was not counteracted by the acute re-administration of the drug.

## 4. Discussion

Sodium channel dysfunction may occur in the setting of both acquired and inherited conditions. During myocardial ischemia, metabolic changes within the myocardium lead to a peak I_Na_ reduction and the consequent slowing of conduction with an increased risk of re-entrant arrhythmias [25]. In heart failure, both a decrease in peak I_Na_ as well as an increase in late I_Na_ may occur secondary to cardiomyocyte remodeling, transcriptional changes, and ionic dysregulation [5,26,27]. Loss-of-function *SCN5A* mutations (associated with Brugada syndrome and cardiac conduction disease) result in reduced peak I_Na_, while gain-of-function mutations (associated with LQT3) disrupt the fast inactivation of the channel, thereby enhancing late I_Na_ [28,29]. Therapies aimed at reducing late I_Na_ have demonstrated beneficial effects in the setting of LQT3, shortening AP duration, attenuating intracellular calcium dysregulation, and preventing arrhythmias [4,30]. In contrast, no pharmacological options are currently available to enhance peak I_Na_ and restore conduction. As a result, the only available approach in patients at risk of life-threatening arrhythmias secondary to the loss of sodium channel function, such as Brugada syndrome, is an implantable cardioverter defibrillator [31]. Hence, there is an unmet need to develop novel therapeutic approaches to safely enhance peak I_Na_. In this paper, we explored whether promoting the trafficking of sodium channels to the cell membrane could be an attractive approach to increase peak I_Na_.

The concept of pharmacological chaperones has been mostly investigated in the field of conformational diseases caused by misfolded proteins secondary to mutations [32]. Pharmacological chaperones, or “pharmacoperones”, are small molecules that serve as scaffolds to stabilize the conformation of misfolded or destabilized proteins, thereby preventing their degradation and promoting their exit from the endoplasmic reticulum and subsequent trafficking to the membrane [33,34]. Their therapeutic potential has been demonstrated for various ion channel disorders, including cystic fibrosis and long QT syndrome type 2 (LQTS2) [33,35,36,37]; in the latter, hERG channel blockers have been shown to rescue the membrane trafficking of mutant channels [37,38,39]. A similar chaperone action has been demonstrated for sodium channel blockers, including mexiletine [16,17,18,40]. However, since most previous studies employed very high, non-therapeutic concentrations of mexiletine (>100 µM), the enhanced trafficking effect is likely countered by the acute blocking effect on the channel. We therefore assessed the beneficial effects of a clinically relevant concentration of mexiletine (10 µM) on I_Na_ and AP characteristics in non-diseased hiPSC-CMs. By incubating hiPSC-CMs for 48 h with mexiletine followed by the removal of the drug prior to patch clamp analysis, we were able to assess the chronic impact of mexiletine (i.e., its chaperone action) without its acute effect. Similar to what we previously been observed for *SCN5A* transfected in HEK293 cells [21], the chronic mexiletine incubation of hiPSC-CMs increased peak I_Na_ without changes in the voltage dependence of (in)activation, suggesting that this “therapeutic” concentration is capable of enhancing the membrane trafficking of normal sodium channels. Indeed, our immunocytochemistry experiments indicate an increased membrane expression of Na_v_1.5 following chronic mexiletine incubation. These observations are in line with a previous study, which employed a radioligand assay using the sodium channel-specific toxin [3H]batrachotoxinin benzoate ([3H]BTXB) and demonstrated that the in vivo administration of mexiletine for 72 h in rats dose-dependently increased the number of sodium channels in cardiomyocytes [14]. The treatment of adult rats with mexiletine for 24, 48, or 72 h (50 mg/kg/day) has also been shown to increase cardiac sodium channel mRNA expression, providing a potential additional mechanism to be further explored [41]. Crucially, in our study, the acute (re-)administration of mexiletine did not affect peak I_Na_ and thus did not counteract its chronic beneficial effect; hence, 10 µM was sufficiently high to enhance membrane trafficking, yet not too high to acutely inhibit the channel. However, the latter may be different during situations of reduced sodium channel availability, for instance, at faster heart rates or during ischemia.

Our results obtained from hiPSC-CMs provide important information beyond previous findings in HEK293 cells, since the latter differ significantly from cardiomyocytes, including the lack of sodium channel interacting proteins. The use of hiPSC-CMs furthermore allowed us to assess the effects of mexiletine on AP characteristics. In line with the observed increase in peak I_Na_, chronic mexiletine incubation significantly increased V_max_ without altering other AP properties; the latter may indicate that the chaperone effect afforded by mexiletine did not affect other ion channels. In contrast, the acute (re-)administration of mexiletine, while not affecting V_max_, did result in a significant prolongation of the AP, which was reversible upon wash-out. Mexiletine has also been shown to decrease both the hERG potassium current and the L-type calcium current [42,43]. Hence, the impact of mexiletine on repolarization will be the net result of its (dose-dependent) effects on different ion currents, warranting a full and detailed exploration in future studies. Interestingly, in our previous study, we showed that the acute administration of 10 µM mexiletine, while acutely inhibiting the mutation-induced late I_Na_ in HEK293 cells, caused only a relatively small but significant decrease in AP duration in *SCN5A*-1795insD hiPSC-CMs [21]. One may speculate that the ability of mexiletine to restore repolarization in *SCN5A*-1795insD hiPSC-CMs was partly mitigated by its inhibition of repolarizing currents. Hence, it may be worthwhile to consider if lower concentrations of mexiletine could still afford the observed beneficial effects on I_Na_, while reducing the risk of potential detrimental effects. This is also of potential relevance in relation to the extra-cardiac side effects of mexiletine (including nausea, abdominal discomfort, tremors, and headaches) that limit its use in some patients who may not tolerate it well [6].

## 5. Conclusions

We demonstrate in this paper that the chronic administration of a clinically relevant concentration of mexiletine increases peak I_Na_ and AP upstroke velocity in non-diseased hiPSC-CMs. These findings extend beyond previous observations that mexiletine can act as a chaperone to restore the trafficking of mutant sodium channels and provide the first evidence that mexiletine can also enhance the membrane expression of normal channels. These results identify mexiletine as a potential therapeutic strategy to enhance peak I_Na_ and cardiac conduction, with a possible future application in arrhythmia disorders associated with reduced peak I_Na_, such as Brugada syndrome. However, further studies are required aimed at minimizing the risk of (extra-)cardiac side effects, including the assessment of dose-response relationships. Moreover, mexiletine has recently been repurposed for myotonia and, as a result, its price has significantly increased [44,45,46]. Hence, it will be important to further investigate the mechanisms underlying the (chaperone) effect of mexiletine on sodium channel trafficking, to facilitate the future development of other, ideally more selective, approaches mimicking its observed beneficial effects.

## Figures and Tables

**Table 1:** Sodium current gating properties in hiPSC-CMs incubated for 48 h with the vehicle or mexiletine.

	Vehicle	Mexiletine
Activation V_1/2_ (mV)	*n* = 15 −33.0 ± 1.6	*n* = 19 −35.1 ± 1.2
*k* (mV) Inactivation V_1/2_ (mV) *k* (mV)	4.3 ± 0.4 *n* = 15 −78.2 ± 1.4 −7.5 ± 0.2	5.1 ± 0.4 *n* = 17 −80.9 ± 1.5 −7.5 ± 0.3

V_1/2_, half-voltage of (in)activation; *k*, slope of the (in)activation curve; *n*, number of cells.

**Table 2:** Effect of acute mexiletine administration on sodium current gating properties in hiPSC-CMs previously incubated for 48 h with the vehicle or mexiletine.

	hiPSC-CMs Incubated with the Vehicle		hiPSC-CMs Incubated with Mexiletine	
	Baseline	Acute Mexiletine Wash-In	Baseline	Acute Mexiletine Wash-In
Activation V_1/2_ (mV)	*n* = 6 −35.0 ± 2.4	*n* = 6 −38.4 ± 2.9	*n* = 7 −36.7 ± 2.1	*n* = 7 −37.4 ± 2.4
*k* (mV) Inactivation V_1/2_ (mV) *k* (mV)	4.5 ± 0.7 *n* = 4 −78.9 ± 2.6 −7.5 ± 0.7	4.0 ± 0.5 *n* = 4 −81.4 ± 1.3 −8.7 ± 0.8	6.5 ± 0.9 *n* = 4 −77.3 ± 1.6 −8.1 ± 0.2	5.4 ± 0.6 *n* = 4 −77.6 ± 1.5 −7.5 ± 0.7

V_1/2_, half-voltage of (in)activation; *k*, slope of the (in)activation curve; *n*, number of cells.

## Data Availability

The data presented in this study are available upon request from the corresponding author.

## References

[1] Veerman C.C., Wilde A.A.M., Lodder E.M. (2015). The Cardiac Sodium Channel Gene SCN5A and Its Gene Product NaV1.5: Role in Physiology and Pathophysiology. Gene.

[2] Chen-Izu Y., Shaw R.M., Pitt G.S., Yarov-Yarovoy V., Sack J.T., Abriel H., Aldrich R.W., Belardinelli L., Cannell M.B., Catterall W.A. (2015). Na+ Channel Function, Regulation, Structure, Trafficking and Sequestration. J. Physiol..

[3] Viswanathan P.C., Balser J.R. (2004). Inherited Sodium Channelopathies s Continuum of Channel Dysfunction. Trends Cardiovasc. Med..

[4] Rivaud M.R., Delmar M., Remme C.A. (2020). Heritable Arrhythmia Syndromes Associated with Abnormal Cardiac Sodium Channel Function: Ionic and Non-Ionic Mechanisms. Cardiovasc. Res..

[5] Moreno J.D., Clancy C.E. (2012). Pathophysiology of the Cardiac Late Na Current and Its Potential as a Drug Target. J. Mol. Cell. Cardiol..

[6] Olleik F., Kamareddine M.H., Spears J., Tse G., Liu T., Yan G.X. (2023). Mexiletine: Antiarrhythmic Mechanisms, Emerging Clinical Applications and Mortality. Pacing Clin. Electrophysiol..

[7] van der Ree M.H., van Dussen L., Rosenberg N., Stolwijk N., van den Berg S., van der Wel V., Jacobs B.A.W., Wilde A.A.M., Hollak C.E.M., Postema P.G. (2022). Effectiveness and Safety of Mexiletine in Patients at Risk for (Recurrent) Ventricular Arrhythmias: A Systematic Review. Europace.

[8] Farkowski M.M., Karlinski M., Pytkowski M., de Asmundis C., Lewandowski M., Mugnai G., Conte G., Marijon E., Anic A., Boveda S. (2022). Mexiletine for Recurrent Ventricular Tachycardia in Adult Patients with Structural Heart Disease and Implantable Cardioverter Defibrillator: An EHRA Systematic Review. Europace.

[9] Alhourani N., Wolfes J., Könemann H., Ellermann C., Frommeyer G., Güner F., Lange P.S., Reinke F., Köbe J., Eckardt L. (2024). Relevance of Mexiletine in the Era of Evolving Antiarrhythmic Therapy of Ventricular Arrhythmias. Clin. Res. Cardiol..

[10] Frommeyer G., Garthmann J., Ellermann C., Dechering D.G., Kochhäuser S., Reinke F., Köbe J., Wasmer K., Eckardt L. (2018). Broad Antiarrhythmic Effect of Mexiletine in Different Arrhythmia Models. Europace.

[11] Mazzanti A., Maragna R., Faragli A., Monteforte N., Bloise R., Memmi M., Novelli V., Baiardi P., Bagnardi V., Etheridge S.P. (2016). Gene-Specific Therapy with Mexiletine Reduces Arrhythmic Events in Patients with Long QT Syndrome Type 3. J. Am. Coll. Cardiol..

[12] Schwartz P.J., Priori S.G., Locati E.H., Napolitano C., Cantu F., Towbin J.A., Keating M.T., Hammoude H., Brown A.M., Chen L.S.K. (1995). Long QT Syndrome Patients with Mutations of the SCN5A and HERG Genes Have Differential Responses to Na+ Channel Blockade and to Increases in Heart Rate: Implications for Gene-Specific Therapy. Circulation.

[13] Moreno J.D., Zhu W., Mangold K., Chung W., Silva J.R. (2019). A Molecularly Detailed NaV1.5 Model Reveals a New Class I Antiarrhythmic Target. Basic Transl. Sci..

[14] Taouis M., Sheldon R.S., Duff H.J. (1991). Upregulation of the Rat Cardiac Sodium Channel by in Vivo Treatment with a Class I Antiarrhythmic Drug. J. Clin. Investig..

[15] Sheldon R.S., Duff H.J., Thakore E., Hill R.J. (1994). Class I Antiarrhythmic Drugs: Allosteric Inhibitors of [3H] Batrachotoxinin Binding to Rat Cardiac Sodium Channels. J. Pharmacol. Exp. Ther..

[16] Valdivia C.R., Tester D.J., Rok B.A., Porter C.-B.J., Munger T.M., Jahangir A., Makielski J.C., Ackerman M.J. (2004). A Trafficking Defective, Brugada Syndrome-Causing *SCN5A* Mutation Rescued by Drugs. Cardiovasc. Res..

[17] Moreau A., Keller D.I., Huang H., Fressart V., Schmied C., Timour Q., Chahine M. (2012). Mexiletine Differentially Restores the Trafficking Defects Caused by Two Brugada Syndrome Mutations. Front. Pharmacol..

[18] Hu R.M., Tester D.J., Li R., Sun T., Peterson B.Z., Ackerman M.J., Makielski J.C., Tan B.H. (2018). Mexiletine Rescues a Mixed Biophysical Phenotype of the Cardiac Sodium Channel Arising from the SCN5A Mutation, N406K, Found in LQT3 Patients. Channels.

[19] Tan B.-H., Valdivia C.R., Song C., Makielski J.C. (2006). Partial Expression Defect for the SCN5A Missense Mutation G1406R Depends on Splice Variant Background Q1077 and Rescue by Mexiletine. Am. J. Physiol. Heart Circ. Physiol..

[20] Nei S.D., Danelich I.M., Lose J.M., Leung L.Y.T., Asirvatham S.J., McLeod C.J. (2016). Therapeutic Drug Monitoring of Mexiletine at a Large Academic Medical Center. SAGE Open Med..

[21] Nasilli G., Yiangou L., Palandri C., Cerbai E., Davis R.P., Verkerk A.O., Casini S., Remme C.A. (2023). Beneficial Effects of Chronic Mexiletine Treatment in a Human Model of SCN5A Overlap Syndrome. Europace.

[22] Verkerk A.O., Wilders R. (2023). Injection of IK1 through Dynamic Clamp Can Make All the Difference in Patch-Clamp Studies on HiPSC-Derived Cardiomyocytes. Front. Physiol..

[23] Dhamoon A.S., Jalife J. (2005). The Inward Rectifier Current (IK1) Controls Cardiac Excitability and Is Involved in Arrhythmogenesis. Heart Rhythm.

[24] Van Putten R.M.E.M., Mengarelli I., Guan K., Zegers J.G., Van Ginneken A.C.G., Verkerk A.O., Wilders R. (2015). Ion Channelopathies in Human Induced Pluripotent Stem Cell Derived Cardiomyocytes: A Dynamic Clamp Study with Virtual IK1. Front. Physiol..

[25] Janse M.J., Wit A.L. (1989). Electrophysiological Mechanisms of Ventricular Arrhythmias Resulting from Myocardial Ischemia and Infarction. Physiol. Rev..

[26] Rivaud M.R., Agullo-Pascual E., Lin X., Leo-Macias A., Zhang M., Rothenberg E., Bezzina C.R., Delmar M., Remme C.A. (2017). Sodium Channel Remodeling in Subcellular Microdomains of Murine Failing Cardiomyocytes. J. Am. Heart Assoc..

[27] Makielski J.C. (2016). Late Sodium Current: A Mechanism for Angina, Heart Failure, and Arrhythmia. Trends Cardiovasc. Med..

[28] Rivaud M. (2020). Functional Modulation of Atrio-Ventricular Conduction by Enhanced Late Sodium Current and Calcium-Dependent Mechanisms in Scn5a1798insD/+ Mice. Europace.

[29] Wilde A.A.M., Amin A.S. (2018). Clinical Spectrum of SCN5A Mutations: Long QT Syndrome, Brugada Syndrome, and Cardiomyopathy. JACC Clin. Electrophysiol..

[30] Portero V., Casini S., Hoekstra M., Verkerk A.O., Mengarelli I., Belardinelli L., Rajamani S., Wilde A.A.M., Bezzina C.R., Veldkamp M.W. (2017). Anti-Arrhythmic Potential of the Late Sodium Current Inhibitor GS-458967 in Murine *Scn5a*-1798insD^+/-^ and Human *SCN5A*-1795insD^+/-^ IPSC-Derived Cardiomyocytes. Cardiovasc. Res..

[31] Cutler M.J., Eckhardt L.L., Kaufman E.S., Arbelo E., Behr E.R., Brugada P., Cerrone M., Crotti L., DeAsmundis C., Gollob M.H. (2024). Clinical Management of Brugada Syndrome: Commentary from the Experts. Circ. Arrhythm. Electrophysiol..

[32] Grasso D., Galderisi S., Santucci A., Bernini A. (2023). Pharmacological Chaperones and Protein Conformational Diseases: Approaches of Computational Structural Biology. Int. J. Mol. Sci..

[33] Hou Z.S., Ulloa-Aguirre A., Tao Y.X. (2018). Pharmacoperone Drugs: Targeting Misfolded Proteins Causing Lysosomal Storage-, Ion Channels-, and G Protein-Coupled Receptors-Associated Conformational Disorders. Expert Rev. Clin. Pharmacol..

[34] Tao Y.X., Conn P.M. (2018). Pharmacoperones as Novel Therapeutics for Diverse Protein Conformational Diseases. Physiol. Rev..

[35] Vauthier V., Housset C., Falguières T. (2017). Targeted Pharmacotherapies for Defective ABC Transporters. Biochem. Pharmacol..

[36] Heard A., Thompson J., Carver J., Bakey M., Wang X. (2015). Targeting Molecular Chaperones for the Treatment of Cystic Fibrosis: Is It a Viable Approach?. Curr. Drug Targets.

[37] Mehta A., Ramachandra C.J.A., Singh P., Chitre A., Lua C.H., Mura M., Crotti L., Wong P., Schwartz P.J., Gnecchi M. (2018). Identification of a Targeted and Testable Antiarrhythmic Therapy for Long-QT Syndrome Type 2 Using a Patient-Specific Cellular Model. Eur. Heart J..

[38] Smith J.L., Reloj A.R., Nataraj P.S., Bartos D.C., Schroder E.A., Moss A.J., Ohno S., Horie M., Anderson C.L., January C.T. (2013). Pharmacological Correction of Long QT-Linked Mutations in KCNH2 (HERG) Increases the Trafficking of Kv11.1 Channels Stored in the Transitional Endoplasmic Reticulum. Am. J. Physiol. Cell Physiol..

[39] O’Hare B.J., Kim C.S.J., Hamrick S.K., Ye D., Tester D.J., Ackerman M.J. (2020). Promise and Potential Peril with Lumacaftor for the Trafficking Defective Type 2 Long-QT Syndrome-Causative Variants, p.G604S, p.N633S, and p.R685P, Using Patient-Specific Re-Engineered Cardiomyocytes. Circ. Genomic Precis. Med..

[40] Zhao J., Ziane R., Chatelier A., O’Leary M.E., Chahine M. (2007). Lidocaine Promotes the Trafficking and Functional Expression of Na(v)1.8 Sodium Channels in Mammalian Cells. J. Neurophysiol..

[41] Duff H.J., Offord J., West J., Catterall W.A. (1992). Class I and IV Antiarrhythmic Drugs and Cytosolic Calcium Regulate MRNA Encoding the Sodium Channel α Subunit in Rat Cardiac Muscle. Mol. Pharmacol..

[42] Gualdani R., Tadini-Buoninsegni F., Roselli M., Defrenza I., Contino M., Colabufo N.A., Lentini G. (2015). Inhibition of HERG Potassium Channel by the Antiarrhythmic Agent Mexiletine and Its Metabolite M-Hydroxymexiletine. Pharmacol. Res. Perspect..

[43] Ono K., Kiyosue T., Arita M. (1986). Comparison of the Inhibitory Effects of Mexiletine and Lidocaine on the Calcium Current of Single Ventricular Cells. Life Sci..

[44] Postema P.G., Schwartz P.J., Arbelo E., Bannenberg W.J., Behr E.R., Belhassen B., Brugada J., Brugada P., Camm A.J., Casado-Arroyo R. (2020). Continued Misuse of Orphan Drug Legislation: A Life-Threatening Risk for Mexiletine. Eur. Heart J..

[45] van den Berg S., van der Wel V., de Visser S.J., Stunnenberg B.C., Timmers L., van der Ree M.H., Postema P.G., Hollak C.E.M. (2021). Cost-Based Price Calculation of Mexiletine for Nondystrophic Myotonia. Value Health.

[46] Postema P.G. (2020). About the Different Faces of Mexiletine. Heart Rhythm.

